# Characterizations of Elephant Endotheliotropic Herpesvirus Type 1A and 4 Co-Infections in Asian Elephant (*Elephas maximus*) Calves

**DOI:** 10.3390/vetsci11040147

**Published:** 2024-03-25

**Authors:** Khajohnpat Boonprasert, Saralee Srivorakul, Natcha Monchaivanakit, Warangkhana Langkaphin, Supaphen Sripiboon, Thittaya Janyamethakul, Channarong Srisa-ad, Thunyamas Guntawang, Janine L. Brown, Chatchote Thitaram, Kidsadagon Pringproa

**Affiliations:** 1 Elephant Hospital, National Elephant Institute, Forest Industry Organization, Lampang 52190, Thailand; 2 Center of Elephant and Wildlife Health, Chiang Mai University, Chiang Mai 50100, Thailand; 3 Center of Veterinary Diagnostic and Technology Transfer, Faculty of Veterinary Medicine, Chiang Mai University, Chiang Mai 50100, Thailand; 4 Department of Large Animal and Wildlife Clinical Sciences, Faculty of Veterinary Medicine, Kasetsart University, Kamphaeng Saen Campus, Nakornpathom 73140, Thailand; 5 Pattara Elephant Farm, Mae Wang, Chiang Mai 50360, Thailand; 6 Save Elephant Foundation, Chiang Mai 50100, Thailand; 7 Faculty of Veterinary Medicine, Chiang Mai University, Chiang Mai 50100, Thailand; 8 Center for Species Survival, Smithsonian National Zoo and Conservation Biology Institute, 1500 Remount Rd., Front Royal, VA 22630, USA; 9 Elephant, Wildlife and Companion Animal Research Group, Chiang Mai University, Chiang Mai 50100, Thailand

**Keywords:** double subtypes, elephant endotheliotropic herpesvirus, infection, Asian elephant

## Abstract

**Simple Summary:**

Elephant endotheliotropic herpesvirus (EEHV) represents a significant viral infection in young Asian elephants (*Elephas maximus*). The EEHV1A genotype predominantly induces EEHV hemorrhagic disease, while the EEHV4 genotype also contributes to hemorrhagic symptoms affecting the gastrointestinal and cardiovascular systems, often leading to fatality in young Asian elephants. Recently, instances of co-infection involving EEHV1A and -4 in Asian elephants have been documented. The present study aims to further elucidate the clinical and pathological lesions in these EEHV1A and -4 co-infection cases, as well as refine treatment protocols.

**Abstract:**

Three cases of double infection with elephant endotheliotropic herpesvirus (EEHV) types 1A and 4 in captive Asian elephants are presented. The first calf was a 4-year-old female that showed initial signs of lethargy and depression. The second calf was a 6-year-old female that displayed signs of depression and diarrhea and died within 48 h of the start of supportive treatment. The third was a 2-year-old male that died suddenly while living with the herd. Necropsies were performed in the first and second elephants, while only a tongue sample was collected from the third calf. EEHV infection was confirmed via quantitative PCR (qPCR) and gene sequencing, revealing double subtypes of EEHV1A and -4 infections. This study describes the hematological and pathological characteristics within the host following double EEHV infection.

## 1. Introduction

Elephant endotheliotropic herpesvirus (EEHV) is the most virulent viral disease in both Asian (*Elephas maximus*) and African (*Loxodonta africana*) young elephants [1,2], and it causes acute hemorrhagic disease (EEHV-HD) with a high mortality. The clinical signs associated with EEHV-HD include lethargy, anorexia, facial edema, tongue cyanosis, and sudden death [3,4,5]. Eight genotypes of EEHV have been identified, including EEHV1A, EEHV1B, and EEHV2-7 [2,6,7,8,9,10,11]. EEHV1 is the most common cause of HD and fatality in young Asian elephants [11]. EEHV4 also causes hemorrhagic symptoms in the gastrointestinal and cardiovascular systems, resulting in fatality in young Asian elephants, and has been documented in Thailand [12] and other countries [13]. There were 103 EEHV cases in Thailand during 2006–2019, including 60 cases of subtype 1A, 6 cases of subtype 1B, 35 cases of subtype 4, and 2 cases of co-infection with subtypes 1A and 4 [13,14]. Moreover, 63 cases of EEHV-HD have been reported in European zoos [15]. To our knowledge, EEHV1A and -4 co-infections in elephants are rarely reported with detailed clinical signs, treatment, gross lesions, and histopathological findings [16]. This study aims to further characterize two prior cases of EEHV1A and -4 co-infections [17,18], in addition to a third case, by describing the clinical signs, pathological findings, and treatment of all three EEHV1A and -4 co-infected calves.

## 2. Case Information

### 2.1. Case No. 1

In Case No. 1, a 4-year-old captive female elephant from the southern part of Thailand showed initial signs of mild depression and anorexia, followed by severe depression, anorexia, facial swelling, and petechial hemorrhage of the tongue. On the afternoon of day 1, the elephant was sent to the Southern Elephant Hospital, National Elephant Institute (NEI), Krabi, Thailand. Dark-yellow urine and brown feces were observed at the hospital. Daily aggressive treatment was immediately initiated by intravenous administration of an antiviral drug (acyclovir, 15 mg/kg BID, Siam Pharmaceutical Co., Ltd., Bangkok, Thailand), antibiotics (cefazolin sodium, 5 mg/kg BID, Nida Pharma Incorporation Co., Ltd., Bangkok, Thailand), anti-inflammatory drugs (dexamethasone 0.1 mg/kg, single shot, Atlantic Laboratories, Co., Ltd., Bangkok, Thailand), fluids, and multivitamin supplements. On day 8, the antiviral was changed to famciclovir (15 mg/kg, Novartis AG, Basel, Switzerland) both orally and rectally, and continued for 10 consecutive days. The calf recovered slightly with normal defecation and urination, and the face swelling was reduced while tongue cyanosis was resolved on day 14. However, on day 19, the elephant presented with signs of lethargy, loss of appetite and a foul odor emanating from the oral cavity. A darkening and softening of the feces (i.e., melena) was observed, with five defecations noted on that day. In the evening of day 19, antibiotics (enrofloxacin 2 mg/kg SID, Bayer AG, Leverkusen, Germany), tranexamic acid (OLIC Co., Ltd., Bangkok, Thailand), and multivitamins were administered and continued for five consecutive days. On days 20–31, the animal was inactive and inapparent. However, on days 32–33, the elephant presented dark watery diarrhea, lethargy, pale mucous membranes, inactivity, anuria and no defecation. Unfortunately, the calf died after 33 days of continuous and aggressive treatment. The body condition score was reduced from 4.5 on day one to 1.5 on day 33 (scale of 1–5, with 1 = very thin and 5 = very fat) before death. Blood samples were collected on days 1, 3, 4, 5, 6, 7, 8, 9, 10, 11, 14, 17, 19, 28 and 34. Unfortunately, due to the limited blood volume that was sampled, only samples obtained from days 3, 5, 8, 11 and 19 were sufficient to test for hematology, blood chemistry profiles and qPCR. The volume of blood samples on other days was sufficient only to perform qPCR tests. EEHV detection was achieved using qPCR [19] at the Animal Diagnostic Center, Faculty of Veterinary Medicine, Kasetsart University, Kamphaeng Saen Campus (FVM-KU-KPS). Briefly, DNA was extracted using NucleoSpin Blood (Macherey-Nagel, Duren, Germany) kits, and samples were screened for EEHV using DNA polymerase (PANPOL) PCR primers. Standard terminase (TER) primers were used for subtype specification of EEHV1A/EEHV1B [6] and EEHV3/EEHV4 [2]. PCR amplification was completed under the following conditions: 98 °C for 3 min, then 40 cycles of 94 °C for 10 s, 60 °C for 30 s, and 72 °C for 30 s, followed by 72 °C for 5 min. Positive EEHV TER PCR products were sequenced using and then analyzed using biological sequence alignment software (Bioedit v. 7.2.6). The phylogenetic relationship was generated by multiple alignments of the nucleic acid program (ClustalX 2.1). Molecular analysis revealed co-infection of EEHV1A and -4 in both the blood and tissue samples.

Serial hematology and blood chemistry analyses were conducted at the Animal Diagnosis Center, Faculty of Veterinary Science, Rajamangala University of Technology Srivijaya, Nakhon Si Thammarat, and are presented in Table 1. Leukocytosis, monocytopenia, anemia and thrombocytopenia were found on days 3–19. The monocyte to heterophil ratios were lower than the reference range during the collection period. Elevations in creatinine, blood urea nitrogen (BUN) and creatinine kinase (CK) were also observed.

A necropsy was performed; tissue and blood samples were collected within 2 h of death to determine the cause of death. Tissue samples were sent to the Veterinary Diagnostic Center, Faculty of Veterinary Medicine, Chiang Mai University (FVM-CMU), for histopathology and immunohistochemistry evaluations. For histopathology, formalin-fixed, paraffin-embedded (FFPE) tissues were cut to a 4 µm thickness and stained with hematoxylin and eosin for routine histopathology. For immunohistochemistry, FFPE tissues were cut to the same thickness on 3-aminopropyl-triethoxysilane-coated slides for immunohistochemical detection of EEHV as previously described [17,22]. Briefly, FFPE sections were deparaffined, rehydrated and antigen retrieved by heating in a microwave (700 watts) for 30 min in citrate buffer (pH 6.0). Next, sections were blocked with 3% hydrogen peroxide (H_2_O_2_) for 5 min at room temperature (RT) followed by blocking hydrophobic bonds and non-specific reactions with 2.5% bovine serum albumin (BSA) for 5 min at RT. Sections were then incubated at 37 °C for 2 h with a primary rabbit polyclonal anti-EEHV glycoprotein B (gB) or anti-EEHV DNApol antibody diluted 1:600 with PBS. Sections were washed three times with PBS and normal goat serum was added and incubated at RT for 30 min. Following this, the sections were incubated with goat anti-rabbit secondary antibody (1:200; Vector Laboratories, Newark, CA, USA) for 45 min at RT, washed and incubated with peroxidase ABC reagent (Thermo Fischer Scientific, Waltham, MA, USA) for 30 min at RT. Finally, 3,3′-diaminobenzidine-tetrahydrochloride-(DAB)-H_2_O_2_ was added, and the sections were incubated for 5 min at RT. Positive tissue reactions resulted in a brown color, which was stopped by tap water, and then counter stained with hematoxylin to determine the positive cells under a light microscope.

**Figure 1:**
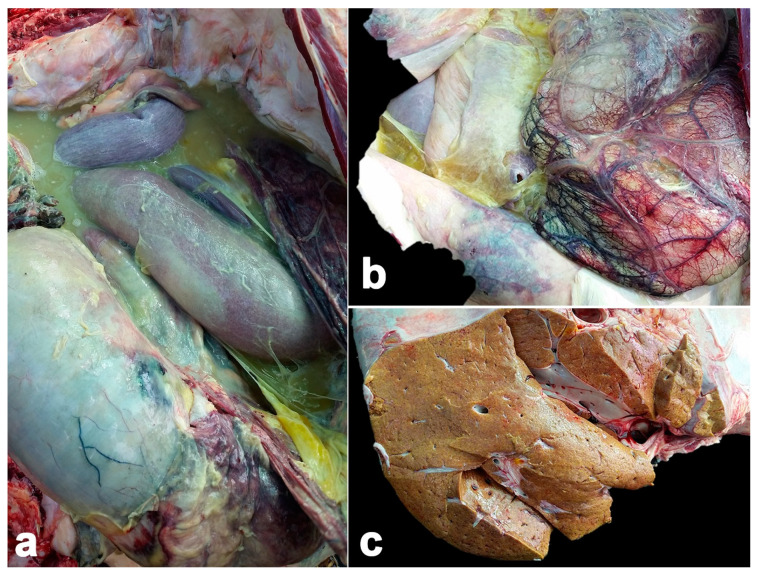
Pathological findings of calf Case No. 1, who died from co-infection of EEHV1A and -4. Multifocal hemorrhage and accumulation of fibrinopurulent exudate was predominantly extended in the abdominal cavity (**a**). A yellowish jelly-like substance, multifocal hemorrhages and congestion were observed in the abdominal serosa (**b**). Liver was pale to yellowish and enlarged (**c**).

Macroscopically, the abdominal cavity presented with accumulation of fibrinopurulent exudate and multifocal hemorrhages (Figure 1). The omentum displayed hyperemia and congestion. The liver was enlarged with a pale to yellowish color. The small intestine was a patchy dark red color and contained dark green- to black-colored ingesta. The spleen was reduced in size, pale and irregularly shaped and presented multifocal hemorrhages, with some purulent exudate plaque. The heart displayed multifocal patchy hemorrhages.

**Figure 2:**
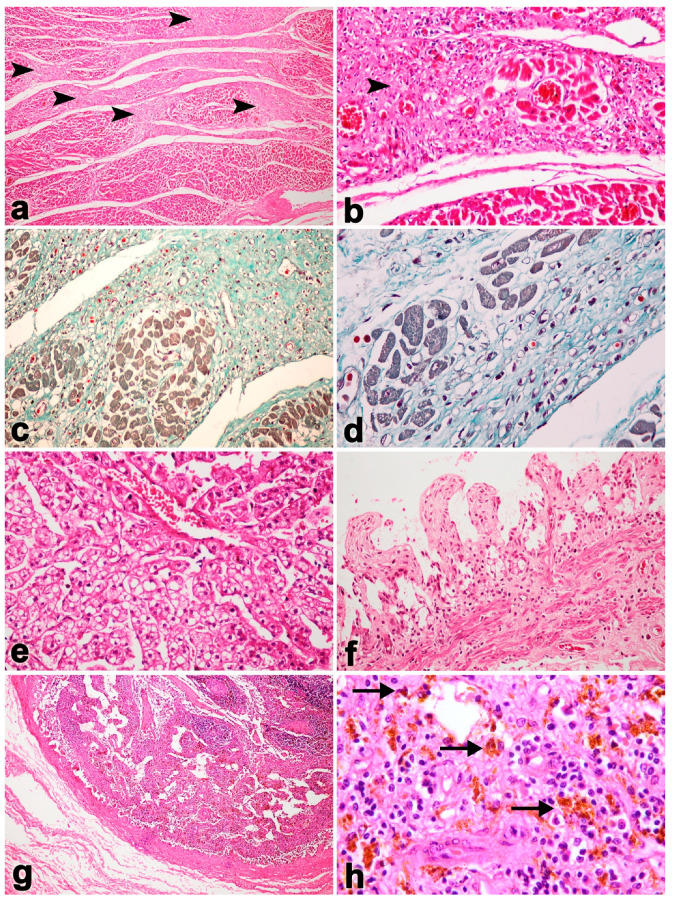
Microscopic findings in Case No. 1. Tissue from the heart shows large, multifocal myocardial fibrosis (arrowhead; (**a**,**b**)). Masson’s trichrome stain revealed large, multifocal areas of myocardial fibrosis and necroses (**c**,**d**). The liver was presented with centrilobular necrosis and fatty degeneration (**e**). Small intestines presented hemorrhages and epithelium necrosis (**f**). There was necrosis of the lymphoid follicles (**g**) and hemosiderosis (arrows) in the lymphoid tissue (**h**).

Microscopically, severe necrosis and hemorrhages were detected in the heart, lung, liver, kidney, spleen, lymph node, stomach and colon (Figure 2). Moreover, bacterial cultures from tissues samples were identified as *Escherlichia coli*, *Streptococcus bovis*, *Streptococcus agalactiae*, *Staphylococcus epidermidis* and coagulase-negative staphylococci in abdominal fluid. *Streptococcus salivarius* was found in the gastrointestinal contents. *E. coli* and *Micrococcus* spp. were found in the mesenteric lymph node samples. Due to practical limitations in the field, anaerobic bacterial culture was not carried out. However, as the carcass displayed pathological lesions resembling those associated with endotoxemia, the abdominal fluids, GI contents, mesenteric lymph nodes and whole blood were collected and subjected to DNA extraction for detection of *Clostridium perfringens* α, β, and ε toxins via multiplex conventional PCR (cPCR), as previously described [23,24]. The result indicates that the *Clostridium perfringens* toxin was positive from wholeblood and spleen samples.

**Figure 3:**
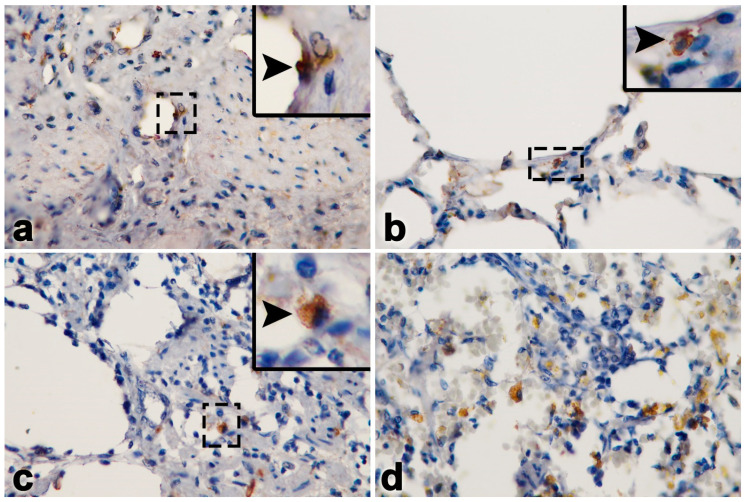
Immunohistochemistry labeling of the EEHV gB positive cells (arrows, inset) in the endothelia and monocytes/macrophages of the heart (**a**), lung (**b**), intestine (**c**) and lymph nodes (**d**) in Case No. 1.

It should be noted that unlike other EEHV-HD cases, elephant Case No. 1 predominantly showed lesions of multifocal fibrosis in the internal organs, including the heart (Figure 2a,b). Masson’s trichrome stain indicated the presence of large, multifocal areas of myocardial fibrosis and necroses (Figure 2c,d). In the liver, centrilobular necrosis and fatty degeneration were observed, while the small intestine exhibited hemorrhages and epithelial necrosis (Figure 2e,f). In addition, hemosiderosis was also predominant and observed in the lymphoid tissue (Figure 2h). Immunohistochemistry labeling of positive cells was performed for EEHV gB in the endothelia and monocytes/macrophages of the heart, lung, intestine and lymph nodes (Figure 3).

In Case No. 1, serial blood samples were available for monitoring viral kinetic changes via qPCR. The results showed a high level of EEHV1A (threshold cycle (Ct) = 28.01) and low level of EEHV4 on day 1 (Ct = 37.14). The EEHV1A viral level started to decrease after treatment, while the EEHV4 viral level was undetectable on day 5 and remained low throughout the treatment period (Table 2). Despite the use of antiviral drugs, the level of EEHV1A increased again on day 28 (no blood sample available from days 18–27) and the animal died on day 34 (Table 2). On the other hand, the viral levels of EEHV4 remained low or undetected throughout the study period (Table 2). Tissue samples tested for the presence of EEHV showed a high level of EEHV1A in most of the internal organs (hearts, liver, intestine, lymph node and feces), with the highest level found in the heart. EEHV4 was detected in the liver, intestine and fecal samples (Table 3).

### 2.2. Case No. 2

In Case No. 2, a 6-year-old female captive elephant presented signs of depression, anorexia, fever and mild diarrhea. Initially, treatment with an antipyretic drug (sodium phenyl dimethylpyrazolone methyl aminomethane sulfate, 3 mg/kg IM, General Drug House Co., Ltd., Pathum Thani, Thailand) and multivitamin supplements was initiated. On the second day, the elephant presented more severe signs such as lethargy, severe depression, anorexia and watery diarrhea. That night, bradypnea, severe depression and bloody diarrhea were observed. At midnight, the elephant lay down, stopped breathing and died 42 h after the initial signs were observed. Only one blood sample was collected at 2 pm on day 2, which was 14 h before death. Necropsy samples were taken the next morning; blood and tissue samples were subjected to EEHV infection tests via qPCR, histopathology and immunohistochemistry. A blood sample was also sent to FVM-CMU for hematological analysis and the data are shown in Table 4. Blood analysis indicated lymphocytopenia, monocytopenia and thrombocytopenia, a low M:H ratio and a high AST (Table 4).

**Figure 4:**
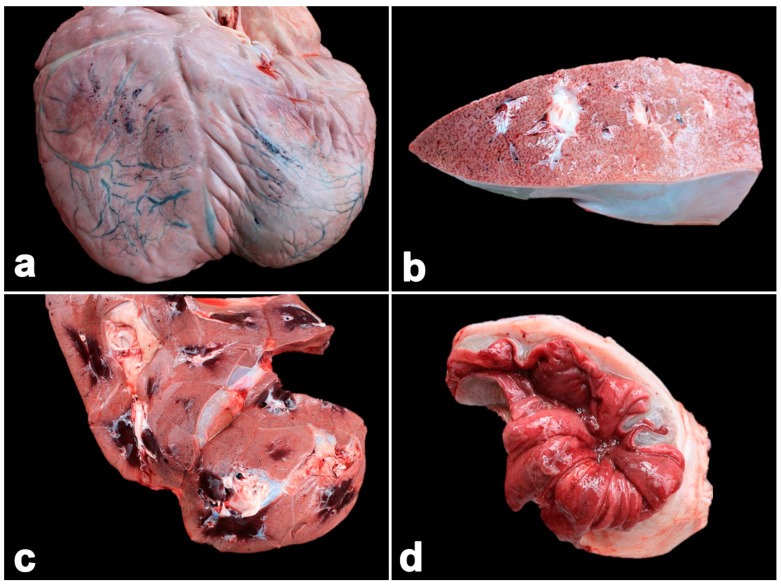
Pathological findings of calf Case No. 2, who died from a co-infection of EEHV1A and -4. Multifocal hemorrhages were observed in the subepicardium (**a**), liver (**b**), kidney (**c**) and intestines ((**d**), cecum). Marked submucosal edema of the intestine with necrosis and hemorrhage of the mucosa were noted (**d**).

**Figure 5:**
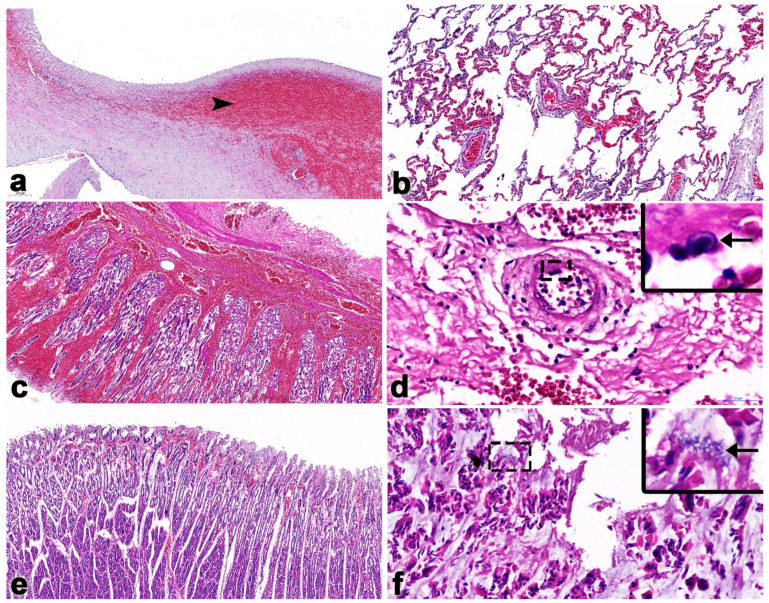
Histopathology findings of calf Case No. 2 that was co-infected with EEHV1A and -4. In the heart, the endocardium showed severe multifocal hemorrhages (arrowhead; (**a**)). The lung exhibited necrotic hemorrhagic pneumonia (**b**). The gastric mucosa contained severe focally extensive hemorrhages (**c**). Intranuclear inclusion bodies (arrow; inset) were found in the endothelium of arteries of the stomach (**d**). There were severe multifocal hemorrhages in the interstitium and perivascular areas of the small intestine (**e**). Intralesional bacterial clumps (arrow; inset) were observed in the intestinal mucosa of the large intestines (**f**).

**Figure 6:**
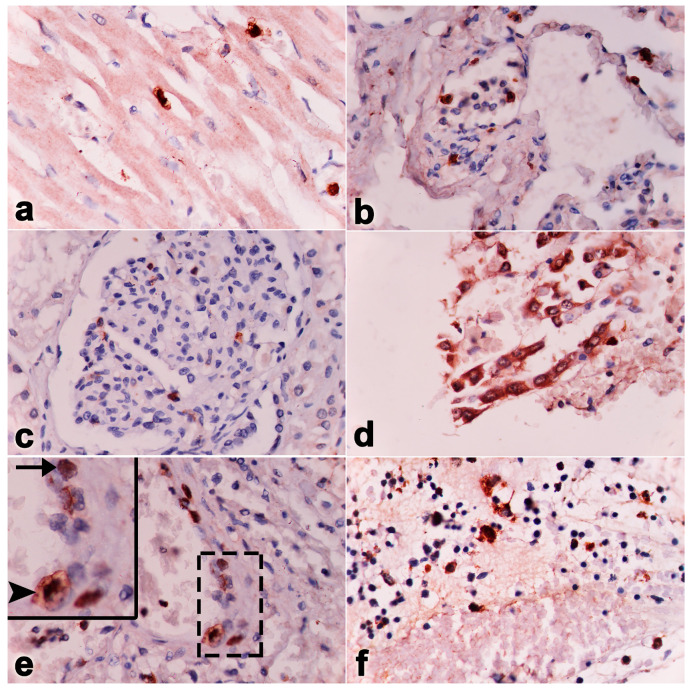
Immunohistochemistry labeling of positive cells in monocytes/macrophages (arrowhead) and endothelia (arrow) for EEHV DNApol in the heart (**a**), lungs (**b**), kidneys (**c**), intestines (**d**), vessels (**e**), and bone marrow (**f**) of Case No. 2.

In the necropsy, the carcass contained generalized purple mucous membranes. The oral cavity contained multifocal ulcerations. The gastrointestinal tract exhibited edema and congestion. The mesenteric and intestinal lymph nodes presented marked edema and enlargement. The liver and kidneys were also hemorrhaged (Figure 4). Multifocal hemorrhages were present in the mucosa of the stomach, small intestine and especially in the cecum (Figure 4). The cecal content was dark red and watery, while the rectum contained numerus cestode-like worms in the necrotic mucosa. The lungs had a red discoloration. Multifocal ecchymotic hemorrhages were present in the epicardium and endocardium. Microscopically, there were severe subacute multifocal hemorrhages in the heart, endocardium, lung, liver, kidney, spleen, stomach and large intestine (Figure 5). Marked bacterial clumps and infiltration by lymphocytes and macrophages were observed in the villi of the large intestines. Intranuclear inclusion bodies were present in the endothelial capillaries of the lung, kidney and stomach (Figure 5). Immunohistochemical findings revealed large numbers of EEHV DNApol immunolabeling-positive cells in the epithelia, endothelia and monocytes/macrophages of the heart, lungs, kidney, liver, intestines and bone marrow (Figure 6).

In Case No. 2, *Clostridium perfringens* was analyzed in the small and large intestinal samples via a similar protocol as described in Case No. 1. The result indicates that the intestinal sample was positive for toxin type β, indicating the possibility of *Clostridium perfringens* co-infection in this case. Moreover, EEHV1A and -4 were confirmed via qPCR testing at FVM-CMU, as described above. 

### 2.3. Case No. 3

In Case No. 3, a 2-year-old captive calf died suddenly while living with its mother and five other elephants. Depression, mild facial edema, and redness of the tongue were observed just 33 h before death. Milk consumption, urination and defecation were still normal. More clinical signs (e.g., lethargy, bradypnea, facial edema, tongue cyanosis and recumbency) were later noticed. The carcass was found in good nutritional condition. However, only the tongue was allowed to be collected for laboratory confirmation of EEHV. The tongue sample was subjected to qPCR at FVM-CMU using the protocol described above. The results were positive for co-infection of EEHV1A and -4. Nevertheless, as necropsy was not carried out, and intestinal contents were not gathered in this particular case; PCR testing for *Clostridium perfringens* infection was not undertaken. 

## 3. Discussion

Co-infections are not common in EEHV cases globally and also in Thailand. To the best of our knowledge, only three cases of EEHV1A and -4 co-infections have been reported, one a juvenile Asian elephant in a European zoo [16] and two cases in Thailand [17,18]. This study further describes in detail the clinical signs, the treatment during infection, the pathological and histopathological findings, and the molecular diagnosis from postmortem examinations of the two previous cases in Thailand plus an additional calf co-infected with EEHV1A and -4.

The calf in Seilern-Moy (2016) died suddenly and exhibited mild depression and lethargy, but no other signs of EEHV-HD [16]. In contrast, the clinical signs in the three elephants in this report were typical of EEHV-HD, such as severe lethargy, facial edema, diarrhea and tongue cyanosis. In Cases No. 1 and 3, lethargy, facial edema and tongue cyanosis were observed on the second day of illness, which are common typical signs of EEHV1, as described in other previous reports [2,11,12,14,25]. In Case No. 2, clinical signs (e.g., facial edema or tongue cyanosis) were not evident; however, bloody diarrhea was observed, which was more likely a clinical sign of EEHV4 infection [14].

There were no hematological results in the EEHV1 and -4 co-infection case reported earlier [16], but in this study, the findings were similar to those in other reports with a single subtype infection [2,11,26,27,28]. Leukocytosis, heterophilia and lymphocytosis in Cases No. 1 and 2 during the initial phase of infection were likely a normal defense mechanism in response to inflammatory processes from viral infection [14,28,29]. However, the hematological results of Case No. 1 later showed lymphocytopenia, monocytopenia and thrombocytopenia, which are hallmarks of progressing EEHV-HD [18,21,26,27,28]. After infection and replication in the endothelia, EEHV-induced endothelial damage occurs throughout the body and increases leakages of plasma from the blood circulation, which causes diffused hemorrhaging and edema of the internal organs [30]. Thus, the hematology changes and clinical signs in this report were similar to other reports, and confirmed that hematological parameters could be useful for differential diagnosis of this viral infection in addition to the diagnosis by molecular confirmation.

Serohemorrhagic fluid in the abdomen, diffuse petechial hemorrhages, extensive edema of all serosal surfaces and multiple hemorrhaging of internal organs are classical pathological findings [2,16,31,32] and similar to the gross findings in Cases No. 1 and 2 that showed widespread hemorrhages, submucosal hemorrhages in the intestine and basophilic intranuclear inclusion bodies in the endothelial cells of the small or medium myocardial arteries and the endocardium. Viral test results in these three cases indicated two patterns of infection. In Case No. 1, both subtypes of EEHV1A and -4 were detected on the first day of infection. However, EEHV1A predominated in the initial phase, followed by co-infection with subtype 4 during the mid-treatment period and up to death. This calf also exhibited soil consumption behavior and presented with *Clostridium perfringens* toxins in luminal contents and organ tissues, similar to a previous report [23]. Although this calf received rapid intensive care, antiviral drugs and supportive treatment following EEHV treatment guidelines [33], the severity of major organ damage caused by EEHV co-infection associated with the *C*. *perfringens* toxin led to fatality in this calf. In contrast, Cases No. 2 and 3 were infected by both subtypes at first clinical presentation, although the rapid onset of death suggested the infection might have been presenting longer than our observation period. This highlights the importance of viral or blood monitoring during the initial phases and intensive treatment until animal recovery or death.

Regarding treatment, antiviral drugs (i.e., famciclovir) are recommended in subclinical and clinical EEHV infections [4,34] and have been used in many successful EEHV treatment cases in western countries. However, in Asian elephant range countries, famciclovir is not available in most pharmacies and is very expensive. Additionally, oral and rectal famciclovir is difficult to administer in untrained calves. Acyclovir is thus a better option for treatment due to its availability in pharmacies and human hospitals, and has been shown to be successful when given intravenously [11,35,36] in a recent study describing its pharmacokinetics and oral bioavailability [37]. Many young calves in Thailand are trained to stand for intravascular treatment without sedation, which is more effective than rectal administration, as the latter method reduces absorption in the large intestine [11].

Additional treatments, such as plasma transfusion should be considered in future cases, which has been shown to increase the survival rate in other cases [11,13]. In range countries, some owners do not allow pathologists to perform necropsies due to cultural beliefs, as in Case No. 3, although a small piece of tongue for molecular and histopathology tests, as in this study, is enough to confirm the initial viral infection [22]. These results are important for further study and to aid in owner awareness education and prevention programs to reduce losses in young Asian elephant populations.

## 4. Conclusions

In this report, we have presented three fatal cases of EEHV1A and -4 co-infections. We described the clinical and pathological changes during EEHV-HD, showing gross and histopathological manifestations indicating organ damage by the virus. These three cases also indicated different results related to rapid diagnosis and antiviral drug therapy. Although all elephants died, the elephant that received rapid antiviral treatment lived longer than the other two elephants that did not. Routine monitoring and surveillance in elephants at risk (i.e., those under 8 years of age) are important to reduce elephant losses until a vaccine is developed to prevent this viral disease.

## Figures and Tables

**Table 1:** Hematological and blood chemistry values of an elephant (Case No. 1) during co-infection with EEHV1A and -4.

Parameter	Normal Range ^a^	Normal Range ^b^	Day 3	Day 5	Day 8	Day 11	Day 19
PCV (%)	30–40	32.25–43.80	24	16	17.5	14	15.3
HGB (g/dL)	11–15	-	9.5	8.9	6.8	5.8	6
RBC (×10^6^ cells/µL)	2.5–5.0	2.17–3.47	2.20	2.06	1.60	1.35	1.4
WBC (cells/µL)	10,000–18,000	2000–4000	23,360	19,550	22,900	15,540	13,650
Heterophil (cells/µL)	1.18–3.57	0.41–1.17	0.30	0.24	0.31	0.28	0.28
Lymphocyte (cells/µL)	5000–8000	6918–13,980	13,548	9970	6412	4972	4360
Monocyte (cells/µL)	2000–4000	2316–5578	2102	1564	2290	2175	1898
Eosinophils (cells/µL)	100–1000	0–535	-	1368	6897	621	546
M:H ratio	1.18–3.57	0.41–1.17	0.30	0.24	0.31	0.28	0.28
Platelet (×10^3^ cells/µL)	200–600	374–554	97	205	377	466	369
Creatinine (mg/dL)	1.0–2.0	-	3.8	6.6	13.6	1.3	3
BUN (mg/dL)	5–20	-	30	35	39	12	63
AST (U/L)	15–35	-	31	35	39	12	63
ALP (U/L)	60–450	-	98	146	176	15	-
GLU (mg/dL)	60–116	-	52	21	64	10	-
TP (g/dL)	6–12	-	6.6	8	8.2	8.1	6.9
CK (U/L)	5–250	-	-	-	-	-	395

^a^ [20] ^b^ [21]

**Table 2:** Threshold cycle (Ct) values associated with viral load levels in blood samples from Case No. 1 throughout the treatment period.

Day Test	Ct (EEHV1A)	Ct (EEHV4)
Day 1	28.06	37.14
Day 3	28.97	35.17
Day 4	29.11	35.87
Day 5	29.00	Undetected
Day 6	31.05	Undetected
Day 7	30.37	37.20
Day 8	30.69	Undetected
Day 9	32.63	38.15
Day 10	32.18	37.27
Day 14	33.05	Undetected
Day 17	33.19	37.08
Day 28	31.34	Undetected
Day 34	31.05	36.83

Note: Days 1–7 treatment with intravenous administration of acyclovir (15 mg/kg, BID). Days 8–18 treatment with famciclovir (15 mg/kg) per oral or rectal routes.

**Table 3:** Threshold cycle (Ct) values associated with viral load levels in samples from Case No. 1.

Tissue	Ct (EEHV1A)	Ct (EEHV4)
Heart	28.67	Undetected
Liver	29.80	33.45
Intestine	32.85	38.08
Lymph node	32.31	Undetected
Feces	25.97	31.15

**Table 4:** Hematological and blood chemistry values of an elephant with an EEHV infection in Case No. 2.

Parameter	Normal Range ^a^	Normal Range ^b^	Day 2
PCV (%)	30–40	32.25–43.80	55
HGB (g/dL)	11–15	-	20.4
RBC (×10^6^ cells/µL)	2.5–5.0	2.17–3.47	4.73
WBC (cells/µL)	10,000–18,000	2000–4000	22,060
Heterophil (cells/µL)	1.18–3.57	0.41–1.17	12,795
Lymphocyte (cells/µL)	5000–8000	6918–13,980	5736
Monocyte (cells/µL)	2000–4000	2316–5578	3530
Eosinophils (cells/µL)	100–1000	0–535	-
M:H ratio	1.18–3.57	0.41–1.17	0.28
Platelets (×10^3^ cells/µL)	200–600	374–554	173
Creatinine (mg/dL)	1.0–2.0	-	1.93
BUN (mg/dL)	5–20	-	20.1
AST (U/L)	15–35	-	54
ALP (U/L)	60–450	-	129
GLU (mg/dL)	60–116	-	-
TP (g/dL)	6–12	-	7.1
CK (U/L)	5–250	-	-

^a^ [20] ^b^ [21]

## Data Availability

No new data were created or analyzed in this study. Data sharing is not applicable to this article.
